# Regulation of DNA Damage Following Termination of Hedgehog (HH) Survival Signaling at the level of the GLI Genes in Human Colon Cancer

**DOI:** 10.18632/oncotarget.586

**Published:** 2012-08-20

**Authors:** Akwasi Agyeman, Tapati Mazumdar, Janet A. Houghton

**Affiliations:** ^1^ Department of Cancer Biology, Lerner Research Institute, Cleveland Clinic, Cleveland, OH

**Keywords:** Hedgehog, GLI1/GLI2 inhibition, GANT61, DNA damage, colon cancer

## Abstract

Transcriptional regulation of the Hedgehog (HH) signaling response is mediated by GLI genes (GLI1, GLI2) downstream of SMO, that are also activated by oncogenic signaling pathways. We have demonstrated the importance of targeting GLI downstream of SMO in the induction of cell death in human colon carcinoma cells. In HT29 cells inhibition of GLI1/GLI2 by the small molecule inhibitor GANT61 induced DNA double strand breaks (DSBs) and activation of ATM, MDC1 and NBS1; γH2AX and MDC1, NBS1 and MDC1 co-localized in nuclear foci. Early activation of ATM was decreased by 24 hr, when p-NBS1^Ser343^, activated by ATM, was significantly reduced in cell extracts. Bound γH2AX was detected in isolated chromatin fractions or nuclei during DNA damage but not during DNA repair. MDC1 was tightly bound to chromatin at 32 hr as cells accumulated in early S-phase prior to becoming subG1, and during DNA repair. Limited binding of NBS1 was detected at all times during DNA damage but was strongly bound during DNA repair. Transient overexpression of NBS1 protected HT29 cells from GANT61-induced cell death, while knockdown of H2AX by H2AXshRNA delayed DNA damage signaling. Data demonstrate following GLI1/GLI2 inhibition: 1) induction of DNA damage in cells that are also resistant to SMO inhibitors, 2) dynamic interactions between γH2AX, MDC1 and NBS1 in single cell nuclei and in isolated chromatin fractions, 3) expression and chromatin binding properties of key mediator proteins that mark DNA damage or DNA repair, and 4) the importance of NBS1 in the DNA damage response mechanism.

## INTRODUCTION

Canonical HH signaling engages the transmembrane receptor PTCH, the intermediary signaling molecule SMO, and the transcriptional regulators of the HH signaling response, GLI. In normal cellular processes, regulation by HH is involved in embryogenesis, tissue patterning, stem cell function, and differentiation [1, 2]. The GLI genes comprise a family of transcription factors that transcriptionally regulate downstream targets in HH-dependent survival. GLI2 appears to be the primary activator of HH signaling, with GLI1 as a transcriptional target of GLI2, which may amplify HH-induced, GLI2-mediated transcription of GLI1 target genes [1, 3-5]; GLI1 and GLI2 induce transcription of overlapping and distinct sets of target genes [1, 3-6], their cooperative roles are critical in HH-dependent survival signaling while their specific roles have been defined only partially [7] GLI1^−/−^ mice have no obvious phenotype [5], in contrast to homozygous GLI2^−/−^ mice which die at birth [6, 8], indicating the critical role of cooperative GLI function in gene regulation and survival.

Dysregulated canonical HH signaling is part of the malignant phenotype of several types of human cancers. Thus, amplification of GLI1 or GLI2, mutations in PTCH or SMO, aberrant gene expression, or upregulated expression of HH ligands, have been identified [1, 7]. Small molecule inhibitors of SMO upstream of GLI have been investigated in preclinical models [9-15], and in the treatment of various types of cancers in humans [14, 16-18]. Those tumors sensitive to SMO inhibitors including basal cell carcinoma [19, 20] and medulloblastoma [16, 21] rely on canonical HH signaling for survival. In other cancer types, SMO inhibitors have demonstrated limited clinical activity (GDC-0449, IPI-926, LDE225; reviewed in [14, 16]). Intrinsic resistance to these agents is frequent [9, 14, 16-18, 22], and acquired resistance to GDC-0449 following initial response by mutation of SMO has been reported in medulloblastoma [23]. In colon cancer, activation of the HH pathway progresses during carcinogenesis and in metastatic disease [11, 24, 25], and is activated in human colon carcinoma cell lines [26, 27] and xenograft models [11], by ligand-dependent and ligand–independent mechanisms. Canonical HH signaling is linked to genomic instability involving inactivation of DNA repair mechanisms, defects in checkpoint activation, and predisposition to development of cancers [28-30]. Chromosome instability is a hallmark of colon cancer, resulting primarily from deregulation of the DNA replication and mitotic spindle checkpoints (reviewed in [31]). We have demonstrated that HH signaling is a critical determinant of cell survival in colon cancer following inhibition of the pathway at the level of the GLI genes downstream of SMO [26, 27, 32, 33]. Non-canonical, oncogene-driven signaling pathways, including activation of the RAS/RAF pathway by genetic mutations in colon cancer, converge on the activation of GLI genes and their downstream targets [7, 22, 34, 35]. Reduced GLI activity in response to the RAS/RAF/MEK/ERK signaling inhibitor U0126 [36, 37] was demonstrated in HT29 cells [33] (mutated B-RAF V600E [38]). This emphasizes that switching off the GLI genes downstream of SMO, that determines HH-dependent transcriptional gene regulation, is critical in terminating HH-dependent survival in cancer cells.

In contrast to SMO, few agents are available that can specifically probe the role of GLI in cell survival. GANT61 was identified in a cell-based screen for small molecule inhibitors of GLI1-mediated transcription. In the original study [39], GANT61 abrogated GLI function in the nucleus, blocked both GLI1- and GLI2- mediated transcription, and inhibited GLI1-DNA binding. We further demonstrated [32] the specificity of GANT61 for GLI1 and GLI2, rapid inhibition of GLI binding to target gene promoters in ChIP analyses, reduced GLI-luciferase activity, and inhibition of transcriptional regulation of target genes after 1 hr exposure to GANT61. A third member of the GLI family, GLI3, is expressed as a cleaved C-terminally truncated form (GLI3R) that silences HH-GLI targets in developmental regulation and embryogenesis [11, 40]. Transient expression of GLI3R repressed GLI1 and GLI2 transcriptional activity in colon cancer cell lines, paralleling the effects of GANT61 [32, 33]. GLI3R transfection not only reduced expression and switched off the function of GLI1 and GLI2, but also induced DNA double strand breaks (DSBs) marked by H2AX nuclear foci, and induced cell death [32]. Following the induction of DNA damage, colon cancer cells accumulated in early S-phase without further progression before becoming subG1 [27]. cDNA microarray gene profiling demonstrated reduced expression of genes engaged in DNA replication, DNA damage signaling, and DNA repair at the G1/S interface [26].

In response to DNA damage, DSBs activate ATM-dependent phosphorylation of H2AX, MDC1, and NBS1. ATM phosphorylates the carboxy-terminal tail of histone H2AX in the vicinity of the break [41,42, 43]. This chromatin modification is crucial for the relocalization of proteins to sites flanking DSBs, and generates foci required to promote efficient repair and sustained DNA damage signaling [44, 45]. MDC1 colocalizes with γH2AX by direct interaction between the C-terminal twin BRCT domains of MDC1 and the γH2AX phospho-epitope [46, 47]. MDC1 also recruits mediators of DNA repair including NBS1 to DNA double strand break sites, and is crucial in nuclear foci to promote sustained DNA damage signaling and repair [41, 46, 47,48, 49]. NBS1 activity in early S-phase is critical for regulation of DNA replication, activation of the intra-S-phase checkpoint, and repair of DNA DSBs [49-56]. NBS1 functions in the evolutionarily conserved MRN (MRE11-RAD50-NBS1) complex in signaling of DSBs within chromatin, in activity at replication forks, and in DNA repair [55-57]. In response to DNA damage, MRN regulates the activity of ATM by direct binding to NBS1 through a C-terminal motif, recruiting ATM to the vicinity of DNA DSBs and stimulating ATM activation [55, 57]. ATM-dependent phosphorylation of NBS1, which occurs at Ser^343^, is then necessary for activation of the MRN complex, localization of MRN to the nucleus, and for recruitment to DNA break sites for repair of damaged DNA [49, 55, 57]. MRE11, which binds at the C-terminus of NBS1, also binds to DNA and provides endonucleolytic activities for DNA processing [57-59]. The MDC1-NBS1 interaction is crucial for the targeting and retention of NBS1 on chromatin flanking DNA DSBs. Following DNA damage signaling, the recognition and processing of DNA damage occur at the onset of S-phase [32, 60].

Using GANT61, cyclopamine and GDC-0449 we have demonstrated the importance of targeting GLI downstream of SMO in termination of HH survival signaling that leads to the induction of cell death in human colon carcinoma cells. Data demonstrate that colon cancer cells resistant to cyclopamine or GDC-0449 remain sensitive to GANT61. Inhibition of GLI1/GLI2 by the small molecule inhibitor GANT61 induces DNA DSBs marked by γH2AX nuclear foci, an ATM-dependent DNA damage signaling mechanism, and activation of MDC1 and NBS1. We have developed a model of DNA damage and DNA repair using GANT61 and explored the mechanisms downstream of GLI1/GLI2 inhibition. Using this model, we have identified the dynamic interactions of the DSB signaling components γH2AX, MDC1 and NBS1 at the level of chromatin in DNA damage signaling upstream of cell death, or in DNA repair. Further, data demonstrate the importance of NBS1 in the outcome of the DNA damage response, following termination of HH survival signaling at the level of GLI, in human colon carcinoma cells.

## RESULTS

### Switching off HH signaling at the level of GLI induces extensive cell death in contrast to targeting SMO upstream of GLI

**Figure 1:**
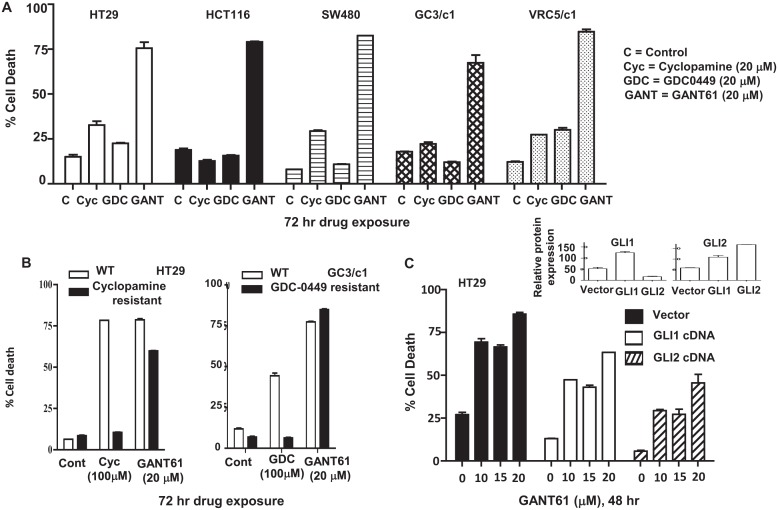
GLI is the critical target in switching off HH survival signaling A: HT29 cells were treated with equimolar concentrations of GANT61 (20 μM), cyclopamine (20 μM) or GDC-0449 (20 μM) for 72 hr. The extent of cell death was determined at the end of drug exposure. B: HT29 or GC3/c1 cells were selected for resistance to cyclopamine or GDC-0449, respectively, by stepwise selection in increasing drug concentrations from 20 μM to 100 μM. Sensitivity of resistant cells to cyclopamine (100 μM), GDC-0449 (100 μM) or GANT61 (20 μM) was examined after 72 hr drug exposure. C: HT29 cells overexpressing GLI1cDNA or GLI2cDNA were examined for their sensitivity to GANT61 at increasing concentrations of 10 μM, 15 μM and 20 μM for 48 hr. Percent cell death was determined. Inset: Relative expression of GLI1 and GLI2 in HT29 cells overexpressing GLI1 (left) or GLI2 (right). Cell death was determined by Annexin V FITC/PI staining and FACS analysis. Data represent the mean +/− SD, n=2.

HT29, HCT116, SW480, GC3/c1 and VRC5/c1 cells were exposed to GANT61 (GLI antagonist), the classic SMO antagonist cyclopamine, or the clinically used SMO inhibitor, GDC-0449, for 72 hr, and their relative efficacies compared at equimolar drug concentrations (20 μM; Figure 1A). Minimal cell death (< 35%) determined by Annexin V/PI staining followed by flow cytometry was obtained in the presence of both SMO antagonists. In marked contrast, GANT61-induced GLI inhibition caused > 85% cell death in all cell lines. To further determine the importance of HH signaling regulation at the level of GLI in cell survival, HT29 or GC3/c1 cells were selected for high-level resistance to cyclopamine or GDC-0449, respectively, at 5-fold higher, supra-physiologic drug concentrations (100 μM). Both HT29 and GC3/c1 cells resistant to SMO inhibitors maintained the high degree of sensitivity to GANT61 (20 μM; Figure 1B). Further, HT29 cells stably overexpressing GLI1 or GLI2 demonstrated reduced sensitivity to GANT61 at all concentrations up to 20 μM examined (Figure 1C).

### NBS1 co-localizes with MDC1 and not γH2AX in nuclear foci; p-NBS1Ser343 is lost from cell extracts following GLI1/GLI2 inhibition

**Figure 2:**
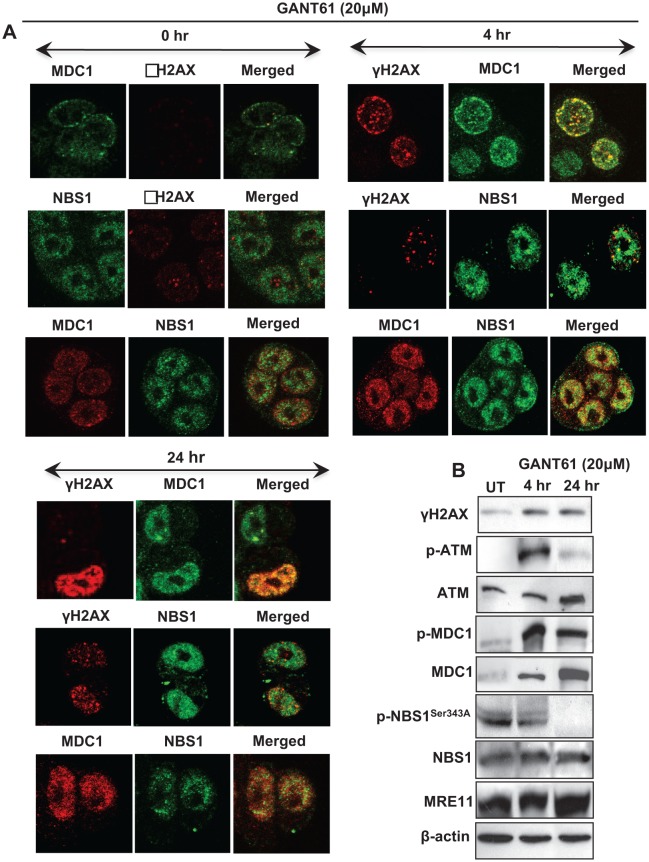
Expression of DNA damage-induced mediator proteins at 4 hr and 24 hr in HT29 cells following inhibition of GLI1/GLI2 by GANT61 (20 μM) A: Localization of γH2AX, MDC1 and NBS1 nuclear foci in single cells and their co-localization was determined after GANT61 administration. After cells were fixed, permeabilized, treated with the respective antibodies, and stained, four-color image acquisition was performed by confocal microscopy and post-processing analysis of the images was as described in Materials and Methods. B: Western analysis of the expression of total and activated forms (γH2AX, p-ATM, p-MDC1, p-NBS1^Ser343^) of mediator proteins was examined after treatment of HT29 cells with GANT61.

HT29 cells were treated for 4 hr or 24 hr with GANT61 (20 μM). Cells were analyzed by confocal microscopy to determine the subcellular localization of γH2AX, MDC1 and NBS1 in cells and in nuclear foci during the induction of early DNA damage at 4 hr [33], and at the G1/S boundary at 24 hr when cells were accumulating in early S [26, 32, 33] (Figure 2A). Alternatively, cells were harvested for western analysis (Figure 2B). Examination at the single cell level revealed γH2AX nuclear foci at 4 hr after GANT61 exposure that were increased in intensity and frequency at 24 hr. MDC1 nuclear foci were also detected at 4 hr and 24 hr following GANT61 and co-localized with γH2AX. In contrast, NBS1 nuclear foci did not co-localize with γH2AX, but were superimposable with MDC1 foci (Figure 2A). Western analysis of HT29 cells at the same time points following GANT61 exposure revealed activation of ATM at 4 hr, that was significantly reduced by 24 hr. MDC1 was activated at both 4 hr and 24 hr. Similar to p-ATM, p-NBS1^Ser343^, phosphorylated by ATM, was present at 4 hr but was significantly reduced in cell extracts at 24 hr (Figure 2B).

### Model of DNA damage and DNA repair

**Figure 3:**
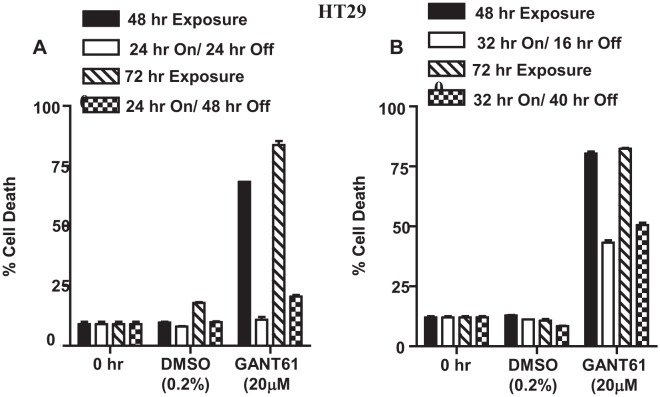
Model of DNA damage and DNA repair in HT29 cells following GLI1/GLI2 inhibition A,B: DNA damage: HT29 cells treated with GANT61 (20 μM) for 48 hr or 72 hr continuous exposure undergo DNA damage upstream of cell death. A: DNA repair: Cells exposed to GANT61 for 24 hr (which induces DNA damage) followed by placing in drug-free medium for 24 hr or 48 hr can be rescued during which time they repair damaged DNA. B: Cells exposed to GANT61 (20 μM) for 32 hr followed by placing in drug-free medium for a further 16 hr or 40 hr can only be partially rescued from cell death. Cell death was determined by Annexin V FITC/PI staining and FACS analysis. Data represent the mean +/− SD, n=2.

To elucidate the mechanisms that influence DNA damage or DNA repair following GLI1/GLI2 inhibition, a model was established in HT29 cells following GANT61 treatment. HT29 cells continuously exposed to GANT61 (20 μM) for ≥ 48 hr undergo DNA damage upstream of cell death (Figure 3). However cells exposed to GANT61 for 24 hr that induces DNA damage can be rescued by placing in drug-free medium, following which time DNA is repaired (Figure 3A). When GANT61 (20 μM) exposure is increased from 24 hr to 32 hr, the ability to fully rescue from GANT61-induced cell death is lost (Figure 3B). Continuous exposure to GANT61, or 24 hr exposure with washout were subsequently used to model DNA damage and DNA repair, respectively.

### Expression of DNA damage signaling molecules during DNA damage and DNA repair

**Figure 4:**
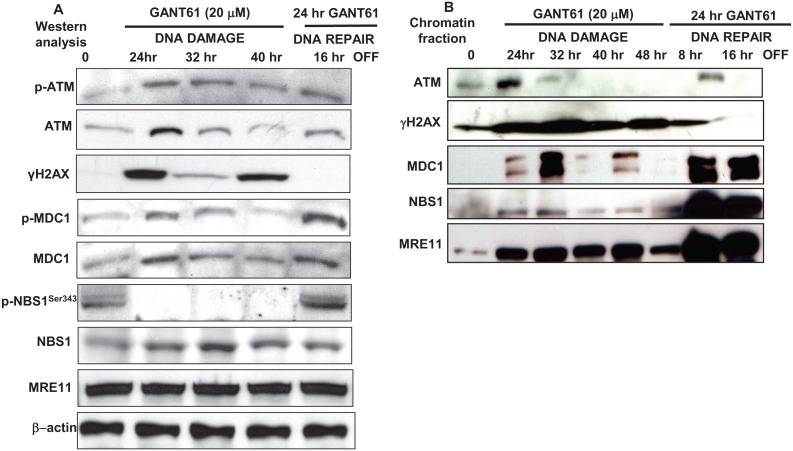
A: The expression of DNA damage-induced mediator proteins was examined following GLI1/GLI2 inhibition for up to 48 hr during continuous exposure to GANT61 (20 μM; DNA damage) or during DNA repair following 24 hr exposure to GANT61 (20 μM) and 16 hr rescue in drug-free medium A: Western analysis. β-actin was used as the loading control. B: Binding of mediator proteins to chromatin following chromatin extraction and western analysis from GANT61-treated HT29 cells as described in Materials and Methods.

Expression of p-ATM and total ATM, γH2AX, p-MDC1, total MDC1, p-NBS1^Ser343^, total NBS1 and MRE11, was examined during DNA damage or during DNA repair in HT29 cells following GANT61 exposure (20 μM). Protein expression was examined for up to 40 hr of continuous exposure to GANT61 (DNA damage), or alternatively following 24 hr GANT61 exposure with a 16 hr washout to allow cells to undergo DNA repair (Figure 4A). Total ATM was expressed at each time point, being more prominent at 24 hr following GANT61 treatment. p-ATM was maintained at the level observed at 24 hr, for up to 40 hr examined. γH2AX, marking DNA DSBs, was expressed for up to 40 hr, being most prominent at 24 hr and 40 hr, however expression in cell extracts disappeared by 16 hr following removal of GANT61. p-MDC1 was detected in cell extracts during the period of DNA damage induced by continuous GANT61 exposure, and expression was significantly increased in the DNA repair phase, while expression of total MDC1 remained relatively constant throughout the experiment. Total NBS1 was present in cell extracts during DNA damage and during DNA repair, however p-NBS1^Ser343^ was undetectable after 24 hr during the DNA damage response, but was re-expressed during DNA repair. MRE11 was expressed at a constant level during both DNA damage and repair phases. GANT61 induction of DNA damage that led to cell death thus correlated with the absence of expression of p-NBS1^Ser343^ from cell extracts.

### Binding of DNA damage signaling molecules to chromatin during DNA damage and DNA repair

Chromatin was isolated from GANT61-treated (20 μM) HT29 cells for up to 48 hr during continuous exposure, or at 8 hr and 16 hr following washout after 24 hr exposure (DNA repair; Figure 4B). ATM was tightly bound to chromatin at 24 hr during GANT61 exposure but was decreased at 32 hr and absent from chromatin at 40 hr. ATM was bound to chromatin during DNA repair, 16 hr after GANT61 was removed from cells. γH2AX was highly expressed at all times during the DNA damage response and tightly bound to chromatin, however after GANT61 was removed at 24 hr, chromatin-bound γH2AX was significantly decreased at 8 hr, and was almost undetectable bound to chromatin at 16 hr during repair of DNA DSBs. MDC1, critical for retention of NBS1 at the sites of DNA breaks, was highly expressed at 32 hr during the DNA damage response (when cells are arrested in early S [32]) and again during DNA repair, 16 hr after GANT61 was removed from cells. In contrast, NBS1 was only weakly detected bound to chromatin between 24 hr and 48 hr during DNA damage, and was highly chromatin bound during DNA repair (Figure 4B). It should be noted that between 24 hr and 40 hr, p-NBS1^Ser343^ was not expressed in cell extracts, but was re-expressed during DNA repair (Figure 4A).

### Confocal microscopy of γH2AX, MDC1 and NBS1 nuclear foci during DNA damage and DNA repair

**Figure 5:**
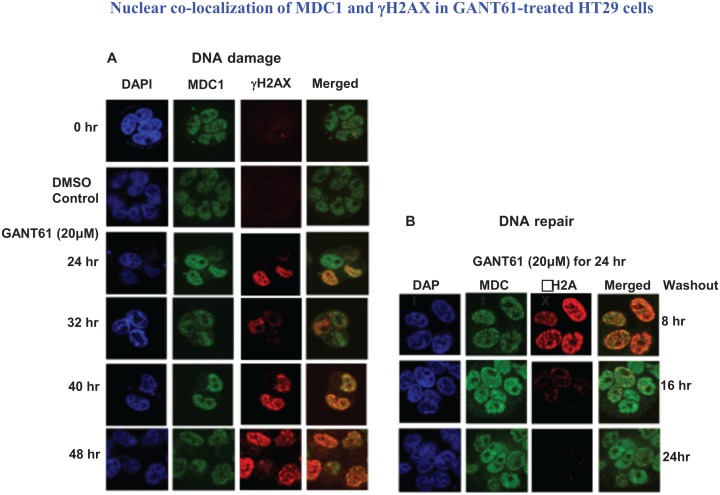
Localization and co-localization of γH2AX and MDC1 nuclear foci during DNA damage or during DNA repair following GLI1/GLI2 inhibition A: HT29 cells were treated with GANT61 (20 μM) for up to 48 hr (DNA damage), or B: for 24 hr followed by incubation in drug-free medium for 8 hr, 16 hr or 24 hr. γH2AX and MDC1 nuclear foci were examined by confocal microscopy as described in Materials and Methods and in the legend to Figure 2.

**Figure 6:**
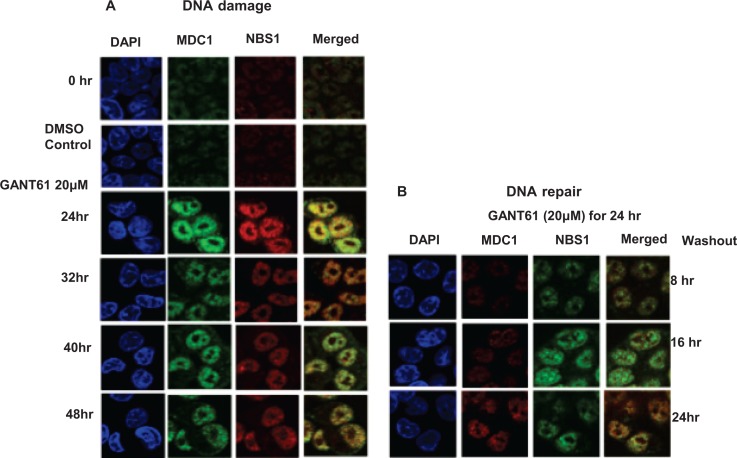
Localization and co-localization of MDC1 and NBS1 nuclear foci during DNA damage or during DNA repair following GLI1/GLI2 inhibition A: HT29 cells were treated with GANT61 (20 μM) for up to 48 hr (DNA damage), or for B: 24 hr followed by incubation in drug-free medium for 8 hr, 16 hr or 24 hr. MDC1 and NBS1 nuclear foci were examined by confocal microscopy as described in Materials and Methods and in the legend to Figure 2.

Co-localization of γH2AX with MDC1 nuclear foci was determined during continuous exposure to GANT61 (20 μM) that marked the sites of DNA DSBs, with retention of MDC1 in nuclei. Nuclear foci were determined in expanded time points for up to 48 hr during continuous exposure to GANT61 (20 μM) and for up to 24 hr following removal of drug after a 24 hr exposure (DNA repair). MDC1 nuclear foci were detected in untreated cells in contrast to γH2AX foci which were significantly less frequent (Figure 5A). Both MDC1 and γH2AX foci were co-localized in nuclei at all times during DNA damage. MDC1 nuclear foci were expressed during DNA repair, correlating with the high degree of binding to chromatin.In contrast, γH2AX nuclear foci rapidly disappeared from nuclei during DNA repair (Figure 5B), which correlates with the disappearance of γH2AX from the chromatin fraction during DNA repair. Co-localization of MDC1 and NBS1 nuclear foci was determined at all times for up to 48 hr during continuous exposure to GANT61 (20 μM; Figure 6A). During DNA repair, MDC1 and NBS1 nuclear foci were also highly abundant and superimposed, at the same time that γH2AX foci were disappearing from nuclei and from isolated chromatin (Figure 6B).

### Contribution of NBS1 and γH2AX to the DNA damage response following GLI1/GLI2 inhibition

**Figure 7:**
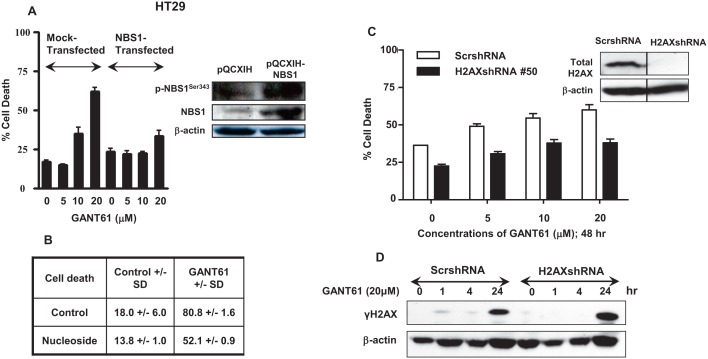
Effect of modulation of NBS1, nucleosides or H2AX during inhibition of HH signaling at the level of GLI A. In transient transfections, HT29 cells were transfected with the retroviral vector pQCXIH alone, or pQCXIH-NBS1 for 24 hr prior to treatment for 48 hr with GANT61 at concentrations of 0 μM, 5 μM, 10 μM, or 20 μM. Cell death was determined. Inset: Total NBS1 and p-NBS1^Ser343^ expression was analyzed by western analysis. B: HT29 cells were treated with 20 μM concentrations of each of adenosine, guanosine, cytidine and thymidine simultaneously with GANT61 (20 μM) for 72 hr, and the extent of cell death determined. C: HT29 cells stably expressing H2AXshRNA or scrambled shRNA were treated with increasing concentrations of GANT61 for 48 hr at which time the extent of cell death was determined. Inset: H2AX knockdown was confirmed by western analysis. D. HT29 cells stably expressing scrambled shRNA or H2AXshRNA were treated with GANT61 (20 μM) for up to 24 hr, and the expression of γH2AX determined by western analysis. β-actin was used as the loading control.

We demonstrated reduced expression of p-NBS1^Ser343^ in cell extracts and reduced binding of NBS1 to chromatin during DNA damage under conditions of GLI1/GLI2 inhibition that led to cell death. Conversely, re-expression of p-NBS1^Ser343^ in cell extracts and avid binding of NBS1 to chromatin during DNA repair correlated with rescue from GANT61-induced cell death. To elucidate the role of NBS1 in regulating the outcome of cellular survival downstream of GLI1/GLI2 inhibition, HT29 cells transiently transfected with pQCXIH-NBS1 or mock transfected with vector alone (pQCXIH) for 24 hr, were treated for a subsequent 48 hr with GANT61 at varied concentrations from 5-20 μM. The influence of NBS1 overexpression on GANT61-induced cell death was determined by Annexin V/PI staining and FACS analysis (Figure 7A). Cell death was inhibited by 30% at the highest concentration of GANT61 examined, demonstrating the critical role of NBS1 in the DNA damage response that regulates cell death following GLI1/GLI2 inhibition. In addition to total NBS1 overexpression, the expression of the active form of NBS1, p-NBS1^Ser343^, was also significantly increased (Figure 7A). We also demonstrated that the nucleosides adenosine, guanosine, cytidine and thymidine administered simultaneously at concentrations of 20 μM, afforded partial protection of HT29 cells from GANT61-induced cell death [27]. Nucleoside rescue from cell death following GLI1/GLI2 inhibition was determined to be ≈ 30% (Figure 7B), similar to the protection afforded following transient transfection and overexpression of NBS1. To determine the role of γH2AX in cell fate following inhibition of GLI1/GLI2 by GANT61, HT29 cells stably expressing H2AXshRNA or scrambled shRNA (ScrshRNA) were treated for 48 hr with GANT61 at doses of 5 μM, 10 μM or 20 μM, and the effect on induction of cell death determined by Annexin V/PI staining and FACS analysis (Figure 7C). Knockdown of H2AX, confirmed by western analysis (Figure 7C Inset) protected cells from GANT61-induced cell death by ≈ 25% at 48 hr. γH2AX expression after GANT61 treatment was further examined by western analysis following suppression of H2AX expression using H2AXshRNA. γH2AX expression was present in cells transduced with the vector control at 1 hr and 4 hr following GLI1/GLI2 inhibition, but not in cells transduced with H2AXshRNA. Under both conditions γH2AX expression was present at 24 hr. H2AXshRNA transduction and reduction in γH2AX expression therefore appeared to delay the detection and recognition of DNA damage following GLI1/GLI2 inhibition. This is consistent with reduced γH2AX binding to chromatin and decreased nuclear γH2AX foci under conditions of cell rescue following GLI1/GLI2 inhibition, and reduction in cell death.

## DISCUSSION

In this study or previously, we have demonstrated that targeting SMO upstream of GLI using the classic SMO inhibitor cyclopamine [27, 33] (employed extensively in preclinical studies), or the clinically used agent GDC-0449, induces minimal cytotoxicity against cell line models of human colon carcinoma exposed at pharmacologically relevant drug concentrations. In contrast, targeting GLI downstream of SMO using the small molecule inhibitor GANT61, which targets both GLI1 and GLI2 transcription, induces extensive cell death in all of these cell line models at equimolar concentrations [27, 33]. Similarly, genetic inhibition of GLI1 and GLI2 using the GLI3 repressor, GLI3R, induces DNA damage, γH2AX expression and nuclear foci, cleavage of caspase-3 and PARP, and cell death, paralleling the effects obtained from pharmacologic targeting of GLI1 and GLI2 [32, 33]. Variable activity of SMO inhibitors has been demonstrated in preclinical models [9-15] and clinically [14, 16-21], in a variety of different types of human cancers. This is due to the predominant dependence of certain types of human cancers on canonical HH signaling (sensitivity; basal cell carcinoma [19, 20], medulloblastoma [16, 21]), or alternatively circumvention of SMO as a therapeutic target in preclinical models and clinically (intrinsic resistance [9, 14, 16-18, 22]) due to activation of GLI by alternate non-canonical, oncogenic signaling pathways [7, 22, 27, 33-35]. In addition, tumors that are initially sensitive to GDC-0449 can develop acquired resistance to SMO inhibitors following prolonged exposure [23]. In the current study, we selected human colon carcinoma cell lines for resistance to supra-physiological concentrations of cyclopamine or GDC-0449 (100 μM), and examined sensitivity of the resistant cell populations to GANT61 (20 μM). Under both conditions, cells maintained high degree of sensitivity to the inhibitor of GLI1/GLI2. Further, HT29 cells engineered to overexpress GLI1 or GLI2 demonstrated reduced sensitivity to GANT61. Collectively, data demonstrate the critical importance of the GLI genes in driving cellular survival in colon cancer cells, and that GLI genes may be activated by ligand-dependent and ligand-independent mechanisms.

Termination of HH signaling at the level of GLI targeted either by pharmacologic or genetic downregulation induces DNA damage [26, 27, 32, 33], which is recognized in early S-phase [26, 32, 60], and marked by γH2AX nuclear foci. DNA replication is inhibited in GANT61-treated cells following GLI1/GLI2 inhibition, where genes including thymidylate synthase, thymidine kinase, topoisomerase2, E2F and DNA polymerases are downregulated in expression [26]. The intra-S-phase checkpoint is activated by DNA DSBs and requires ATM [60]. This checkpoint inhibits progression through S-phase, initiation of late origins of replication [61, 62], and stabilizes stalled replication forks [63]. GANT61-treated cells undergo intra-S-phase checkpoint activation at 24 hr, characterized by phosphorylation of Cdc25A on Ser123 (which targets this phosphatase for proteasomal degradation), activation of Cdk2 is inhibited (decreased ^Tyr15^p-Cdk2), and cyclin E accumulates [32, 33]. We have previously reported accumulation of HT29 cells in early S following GLI1/GLI2 inhibition and activation of an intra-S-phase checkpoint that cannot be sustained, with cells becoming subG1 without further progression through S-phase [32, 33]. In the current study we have identified the molecular interactions between the different signaling molecules involved in DNA damage subsequent to inhibition of GLI1/GLI2 function.

In response to DNA damage, an evolutionarily conserved MRN complex (MRE11, RAD50 and NBS1) regulates the activity of ATM by direct binding of ATM to NBS1, thereby recruiting ATM to the vicinity of DNA DSBs and stimulating ATM activation [55, 57]. NBS1 functions in an evolutionarily conserved complex with MRE11 and RAD50 (MRN) in several cellular contexts including the repair of DNA DSBs (non-homologous end joining, homologous recombination), recognition and signaling of DSBs within chromatin, activity at replication forks [55-57], and is active in early S-phase but not in mid or late S-phase [49-52,53-56]. NBS1 is essential for activation of the intra-S-phase checkpoint in early S to allow repair of DNA damage [54, 56, 57]. In response to DNA damage, MRN regulates the activity of ATM by direct binding of ATM to NBS1, thereby recruiting ATM to the vicinity of DNA DSBs and stimulating ATM activation [55, 57]. ATM-dependent phosphorylation of NBS1 at Ser343 is then necessary for activation of the MRN complex and for the recruitment of MRN to DNA break sites for repair of damaged DNA[49, 55]. MRN imparts three key functions: 1) DNA binding and processing, 2) DNA tethering to bridge DNA over short and long distances, and 3) activation of the DSB response and checkpoint signaling. NBS1 is essential for localization of MRN to the nucleus and for binding to DNA [57]. MRE11, which binds at the C-terminus of NBS1, and also binds to DNA, provides endonucleolytic activities for DNA processing [57-59]. Finally, RAD50 provides regulatory ATPase and adenlyate kinase activities [57]. NBS1 functions in regulation of MRN activity, where the endogenous concentration of active, phosphorylated NBS1 is a critical regulatory factor [49, 64-67]. Our data demonstrate that upon blocking GLI1/GLI2 activity, DNA damage is induced during which the steady state level of p-NBS1^Ser343^ is significantly reduced by 24 hr post-treatment, concurrent with reduced chromatin-associated NBS1 levels. These observations suggest limited NBS1-mediated DNA repair events following GANT61-mediated termination of GLI1/GLI2 function. In response to DNA damage, DSBs activate ATM to phosphorylate the carboxy-terminal tail of histone H2AX in the vicinity of the break [41], a well recognized marker of DNA DSBs [42, 43]. This chromatin modification is crucial for the relocalization of several proteins to sites flanking DSBs, generating foci that are required to promote efficient repair and sustained DNA damage signaling. MDC1 co-localizes with γH2AX [46, 47], and recruits additional mediators of DNA repair including the MRN complex [48, 49]. Although early reports suggested that the N-terminal FHA-BRCT domains of NBS1 enabled phosphorylation-dependent interaction with γH2AX to retain NBS1 at the sites of DSBs, it now appears that the retention of NBS1 is mediated by binding through a specific region of MDC1 (at residues 219-455) that contains six SDTDXD/E (STD) clusters, and which are constitutively phosphorylated by CK2 in unperturbed cells [47, 56, 68-70]. This MDC1-NBS1 interaction via a phospho-dependent mechanism appears crucial for the targeting and retention of NBS1 on chromatin flanking DNA DSBs, and occurs in GANT61-induced chromatin modifications, as we have demonstrated by confocal microscopy in human colon carcinoma cells. Thus, NBS1 co-localized in nuclear foci with MDC1 but not γH2AX, and γH2AX co-localized with MDC1 to facilitate DNA damage signaling. It is evident that γH2AX and p-MDC1 were activated during DNA damage, while p-NBS1^Ser343^ was significantly decreased in cell extracts by 24 hr, in parallel with decreased availability of p-ATM.

We developed a model of DNA damage and DNA repair, where specific mechanisms could be determined in the same model system following GLI1/GLI2 inhibition with GANT61. HT29 cells under continuous GANT61 (20 μM) exposure for 48 hr undergo DNA damage that leads to cell death; cells exposed to GANT61 for 24 hr but not 32 hr were rescued by placing in drug-free medium, during which time they repaired damaged DNA. By 32 hr of continuous GANT61 exposure, cells had arrested in early S but could not progress [26, 32]. μH2AX and p-MDC1 were expressed during continuous GANT61 (20 μM) treatment. During DNA repair, γH2AX expression was significantly reduced, while p-MDC1 expression was increased. Total NBS1 and MRE11 were expressed in GANT61-treated cells during both DNA damage and DNA repair. In contrast, p-NBS1^Ser343^ expression was significantly reduced at ≥ 24 hr during continuous GANT61 exposure, along with lower levels of p-ATM (in comparison to the enhanced activation at 4 hr [32]), but was re-expressed during DNA repair. Upon examination of isolated chromatin fractions from GANT61-treated cells, ATM was highly bound in chromatin fractions at 24 hr during continuous exposure but not at later times, and at 16 hr during DNA repair. γH2AX was tightly bound to chromatin during DNA damage, and released during DNA repair. MRE11 was strongly bound to chromatin during DNA damage and during DNA repair. MDC1 was strongly bound to chromatin during the period following GLI1/GLI2 inhibition when cells were accumulated in early S-phase (24 hr, 32 hr [32]), and also during DNA repair when the presence of γH2AX in chromatin fractions was significantly decreased. However NBS1 binding was limited when cells were undergoing DNA damage, paralleling the loss of p-NBS1^Ser343^ from cell extracts, and became strongly bound during DNA repair. The findings obtained for γH2AX, MDC1 and NBS1 were confirmed in single cells by confocal microscopy. Thus, co-localization of γH2AX and MDC1, MDC1 and NBS1, disappearance of γH2AX as DNA DSBs were repaired after removal of GANT61, with maintenance of NBS1 and MDC1 co-localization, were determined in nuclear foci. Data suggest that p-NBS1^Ser343^ required for appropriate intra-S-phase checkpoint formation and sustained activation may be limiting in the processing of DNA damage signaling at the intra-S-phase checkpoint upstream of cell death, following GLI1/GLI2 inhibition.

To further test this hypothesis, HT29 cells were transiently transfected with pQCXIH-NBS1 for 24 hr prior to treatment with varied concentrations of GANT61 (5 μM, 10 μM, 20 μM) for 48 hr. Overexpression of NBS1 and its activated form, p-NBS^Ser343^, significantly inhibited GANT61-induced cell death, confirming the critical role of NBS1 in regulating the DNA damage response downstream of GLI1/GLI2 inhibition. This was similar to the protection from DNA damage afforded by nucleosides that rescued cells from GANT61-induced cell death, when DNA replication and key regulatory genes were downregulated [26, 32]. To determine the contribution of γH2AX to DNA damage signaling upstream of cell death, HT29 cells stably expressing H2AXshRNA were treated with varied concentrations of GANT61 for 48 hr. Partial decrease in cell death was determined under these conditions, and γH2AX expression was decreased within the first few hours of GANT61 treatment, but was restored at 24 hr. These data together with data derived by western analysis and confocal microscopy suggest that the primary function of γH2AX is in the recognition of DNA DSBs and in DNA damage signaling, and that suppression of H2AX expression causes delay in the recognition of DNA damage.

In summary we have demonstrated that the processing of DNA damage following inhibition of HH signaling at the level of GLI provides a specific rank order of activation of ATM and downstream target genes that recognize DNA damage in early S-phase. The activation of a transient intra-S-phase checkpoint is characterized by limited availability of p-NBS1^Ser343^ in cell extracts and limited binding of NBS1 to chromatin via MDC1 during the critical phase at which colon carcinoma cells accumulate in early S-phase and attempt to repair damaged DNA. Phosphorylated NBS1 reappears in cell extracts during DNA repair and NBS1 is strongly bound to chromatin via MDC1. The function of γH2AX appears to be predominantly in the recognition of DNA damage, and in recruitment of MDC1 to sites of DNA breaks marked by H2AX. It is evident that when MDC1 is recruited to γH2AX DNA break sites, this mediator protein remains bound to chromatin, as does NBS1, while γH2AX disappears from chromatin when DSBs are repaired. It is evident that the drivers of HH signaling, the GLI genes, are critical to the survival of human colon carcinoma cells, and that DNA damage signaling downstream of GLI1/GLI2 inhibition is a critical regulator of cell death.

## MATERIALS AND METHODS

### Antibodies and Chemicals

GANT61 was obtained from Alexis Biochemicals (Farmingdale, NY), cyclopamine from Toronto Research Chemicals (Toronto, ON), and GDC-0449 from JS Research (Boston, MA). Drugs were dissolved in DMSO (0.2%) and stored at -20°C. Antibodies: Anti-NBS1, anti-γH2AX and anti-Mre11 monoclonal primary antibodies were obtained from Cell Signaling Technology (Danvers, MA). Anti-MDC1 polyclonal antibody and anti-p-NBS1^Ser343^ and p-MDC1 monoclonal antibodies were obtained from Abcam Ltd (Cambridge, MA). Peroxidase–conjugated goat anti-rabbit or goat anti-mouse secondary antibodies were from Santa Cruz Biotechnology (Santa Cruz, CA).

### Cell culture and transfections

GLI1cDNA, GLI2cDNA, NBS1cDNA and H2AXshRNA transduction: HT29, HCT116 and SW480 cells were obtained from ATCC. GC3/c1 and VRC5/c1 cells were established in our laboratories from a human colon adenocarcinoma xenograft model [71]. Cell lines were routinely verified by morphology, growth characteristics, and response to cytotoxic agents (Annexin V/propidium iodide (PI) staining). cDNA microarray gene profiles were also characteristic. Cell lines were verified biannually to be mycoplasma-free. Cells were routinely maintained in the presence of folate-free RPMI 1640 medium containing 10% dFBS and 80 nM [6RS]5-methyltetrahydrofolate. Full length GLI1 and GLI2 delta N (N-terminus deleted constitutively active mutant; hereon referred to as GLI2) in pBabe-Puro vector were a kind gift from Dr. Graham W. Neill (Barts and The London School of Medicine and Dentistry, Queen Mary University of London). pQCXIH-NBS1 was a kind gift from Dr. Stephen P. Jackson (Cambridge, England). H2AXshRNA and scrambledshRNA in the pGFP-V-RS vector were obtained from Origene (Rockville, MD). GLI1cDNA, GLI2cDNA, H2AXshRNA and ScrshRNA were subcloned into pBabepuro. HT29 cells stably expressing each construct were generated in the presence of a selection antibiotic, puromycin (1 μg/ml) for 2 weeks, and gene expression confirmed. HT29 cells were transiently transfected with pQCXIH-NBS1 or vector control using lipofectamine-2000 following the manufacturer's protocol. At 24 hr post-transfection, cells were treated with varied concentrations of GANT61 (5μM, 10μM, 20μM) for 48 hr. Cell death was determined by Annexin V/PI staining followed by flow cytometry.

### Annexin V/PI staining and flow cytometric analysis

Annexin V/PI staining and flow cytometric analysis was performed as described previously [27, 33]. Briefly, cells were treated, in duplicate, as described in the figure legends, after which they were collected by trypsinization and incubated with Annexin V FITC (BD Biosciences) and PI (Sigma) prior to analysis using a FACSCalibur flow cytometer. Raw data were analyzed using CellQuest software.

### Western blot analysis

Total cellular lysates were prepared using modified RIPA lysis buffer (Cell Signaling Technology, Danvers, MA). Total protein (54 μg) from cell lysates was loaded and resolved on 10% or 5% SDS-PAGE gels, dependent on protein size. Proteins were transferred onto polyvinylidene difluoride membranes, blocked (5% nonfat dry milk; 1 hr), washed and incubated with primary antibody overnight at 4°C, and with secondary antibody for 1 hr and prior to development.

### Chromatin isolation

HT29 human colon carcinoma cells either mock-treated or treated with GANT61 (20 μM) for varying time periods, washed × 2 with ice-cold 1x PBS, and collected as described [72]. Cell pellets (≈ 2 × 10^6^ cells) were resuspended in 200 μl extraction buffer (50 mM Hepes, pH 7.5, 150 mM NaCl, 1 mM EDTA, supplemented with 0.1% Triton X-100, protease inhibitor mixture tablets [Roche Diagnostics] and phosphatase inhibitors [10 mM NaF, 10 mM β-glycerophosphate, 1 mM sodium orthovanadate, 1 mM cantharidin]; Sigma) for 15 min on ice for the first extraction. Cells were subsequently centrifuged at 14,000 × g, 3 min, followed by a second extraction on ice for 15 min with 200 μl of fresh extraction buffer. The extract was centrifuged at 14,000 × g, 3 min, 4°C. The supernatant was pooled with that from the first extraction. The pellet was further incubated in 200 μl of extraction buffer without Triton and supplemented with 200 μg/ml RNase A (Sigma, St. Louis, MO) for 30 min at 25°C with agitation. Following centrifugation for 3 min at 14 000 × g, the insoluble pellet was resuspended in 1x PBS containing 1% SDS and heated for 10 min at 100°C. Samples were sonicated for 10 sec and denaturing loading buffer (60mM Tris-HCl, pH 6.8, 30% glycerol, 10% SDS, 0.6M DTT, 0.012% bromophenol blue) was added to make a 1 × final concentration. Samples were boiled for 5 min and equal sample volumes of each fraction obtained from equivalent numbers of cells were applied to SDS/PAGE for western blotting.

### Confocal microscopy

Cells were plated at a density of 50,000/well in 6-well plates on coverslips and allowed to attach overnight. The following day, media was removed and the cells were treated in the absence or presence of GANT61 (20μM) for various times with or without drug washout after 24 hr. The coverslips were removed and placed in a humidity chamber for fixation, permeabilization and staining. Images were collected using an HCX Pl Apo 63X, 1.4NA oil immersion objective with Zoom 2 on a Leica SP2 confocal microscope with spectrophotometric detection (Leica Microsystems, GmbH, Wetzlar, Germany). Four-color image acquisition was performed and analysis of images was conducted using Image-Pro Plus software (Media Cybernetics, Inc., Bethesda, MD).
